# Preparation of Ceramic Fiber Threads with Enhanced Abrasion Resistance Performance

**DOI:** 10.3390/ma17030599

**Published:** 2024-01-26

**Authors:** Xueying Zhang, Feng Hou, Haiyan Du, Liwen Yan, Anran Guo, Xiaohui Ma, Jiachen Liu

**Affiliations:** Key Laboratory of Advanced Ceramics and Machining Technology of Ministry of Education, School of Materials Science and Engineering, Tianjin University, Tianjin 300072, China; zxy_92@163.com (X.Z.); hydu@tju.edu.cn (H.D.); lwyan@tju.edu.cn (L.Y.); arguo@tju.edu.cn (A.G.); xiaohuima@tju.edu.cn (X.M.)

**Keywords:** ceramic fiber, coating, abrasion resistance performance, waterborne polyurethane

## Abstract

Ceramic fiber thread is one of the key components in flexible external thermal insulation blankets, and it has been applied in various fields as a flexible ceramic fibrous material with excellent deformability and high-temperature resistance. However, ceramic fiber threads are often subjected to reciprocating friction motion at specific bending angles, making them highly susceptible to abrade and fracture. Enhancing the abrasion resistance performance of ceramic fiber threads under bending conditions is the future trend and remains a significant challenge. Hence, we design and construct a novel polyurethane-modified coating on the ceramic fiber threads to improve their abrasion resistance performance. The effects of the types and concentrations of modifiers on the microstructure, abrasion resistance property, and tensile property of ceramic fiber threads are systematically investigated. The ceramic fiber threads, after modification with hexamethylene diisocyanate waterborne polyurethane (HDI-WPU) with a concentration of 3%, exhibit excellent abrasion resistance properties. The number of friction cycles at fracture of the modified ceramic fiber thread is more than three times, and the tensile strength is more than one and a half times, that of the original ceramic fiber thread, demonstrating the great potential of the HDI-WPU modifier for enhancing the abrasion resistance performance of ceramic fiber threads.

## 1. Introduction

Aerospace vehicles are a focus of attention in the aerospace industry. However, they face a severe aerodynamic heating problem during high-speed flight [1,2,3]. To block heat flux transfer inward and ensure safe service in extreme environments, it is of great significance to improve the comprehensive performance level of the existing thermal protection materials [2,4,5,6,7]. As a commonly used large-area ceramic passive thermal protection material for aerospace vehicles, flexible external thermal insulation blankets have broad application prospects due to their excellent insulation performance, thermal shock resistance, and compression-rebound performance [8,9,10]. The structure of flexible external thermal insulation blankets mainly consists of ceramic fiber felts, ceramic fiber fabrics, and ceramic fiber threads [11,12,13]. As one of the key components, ceramic fiber thread connects and fixes the structures of the various parts of the flexible external thermal insulation blankets and constructs an important flexible skeleton.

Ceramic fiber thread is typically obtained by twisting continuous ceramic fiber bundles [14]. Owing to its good deformability and high-temperature resistance as a flexible ceramic fiber material [15,16,17,18,19], it has garnered significant attention in effectively connecting and securing high-temperature thermal protection and thermal sealing materials [20]. In the application process, ceramic fiber threads often need to undergo reciprocating friction movements within the processing equipment or in the internal structure of the materials they connect. Due to the combined effect of friction and tensile stress, ceramic fiber threads are prone to galling or abrasion, ultimately resulting in fracture failure. Furthermore, the reciprocating friction motion usually occurs at a certain angle rather than in a direction parallel to the axial direction, which exacerbates the stress concentration and abrasion in the bending area of the ceramic fiber thread. It significantly affects reliability, restricts the development of ceramic fiber threads, and threatens the safety of aerospace vehicles in service. Therefore, improving the abrasion resistance performance of ceramic fiber threads is a pressing concern. However, research on the abrasion resistance performance of ceramic fiber threads is still lacking, and studies exploring the abrasion resistance performance of materials at a given bending angle are even rarer. Thus, enhancing the abrasion resistance performance of ceramic fiber thread at specific bending angles remains a huge challenge.

While ceramic fiber threads differ from the raw materials of natural fiber yarns and most organic fiber yarns, they share similar yarn structures [21]. Thus, strategies to improve the abrasion resistance performance of yarns made from different raw materials can provide valuable insights. To date, researchers have employed diverse methods to enhance the abrasion resistance performance of yarns made from cotton [22], polyester [21], carbon fibers [23], and fabrics consisting of polyester–cotton blends, polyester, and other materials [24]. These methods include altering the spinning process, adjusting fabric structures, and applying resin finishing. Inspired by these approaches, two primary methods can be used to enhance the abrasion resistance performance of ceramic fiber threads. The first strategy is post-treatment surface modification, which involves regulating the surface structure and properties of the ceramic fiber threads. This strategy can be achieved by constructing an abrasion-resistant coating with strong interfacial adhesion on the surface of the ceramic fiber threads, endowing them with good abrasion resistance performance and a longer service life. The second strategy is secondary processing optimization, which focuses on adjusting and optimizing the organizational structure of the original ceramic fiber threads [21]. This strategy includes compounding with yarns made of other materials and modifying the number of strands or twists in the ceramic fiber threads [25]. The former strategy is universal, facile, and cost-effective, while the latter is highly specialized and demands advanced equipment, increased operational complexity, and higher costs. Consequently, the post-treatment surface modification strategy holds significant advantages. 

For post-treatment modification, the preparation process of the abrasion-resistant coatings and the selection of the abrasion-resistant modifiers are crucial. Common preparation processes for coatings include the sol-gel method [26], plasma-enhanced chemical vapor deposition method [27], spray-coating method [28], and dip-coating method [29]. Given the simplicity and cost-effectiveness of the preparation process and the wide range of available dipping modifiers, the dip-coating method is expected to be an effective and suitable preparation process to enhance the surface abrasion resistance performance of ceramic fiber threads. The available abrasion-resistant modifiers for threads mainly include organic and inorganic modifiers [30]. Due to the long-term use of ceramic fiber threads in high-temperature environments, the modifiers must fulfill the dual requirements of improving abrasion resistance while preserving mechanical properties at high temperatures. Organic modifiers typically exhibit stability at room temperature and strong interfacial adhesion with the ceramic fiber thread matrix, and can be completely volatilized and removed within an oxidative environment between 300 °C and 600 °C without adversely affecting the performance of the ceramic fiber thread. Inorganic modifiers are stable under high temperatures [31,32,33], but their interfacial adhesion with the ceramic fiber thread matrix is generally weak at room temperature, making it challenging to effectively protect the ceramic fiber thread matrix during repeated reciprocating friction. Therefore, organic modifiers have emerged as highly promising options for improving the abrasion resistance performance of ceramic fiber threads. Among diverse organic abrasion-resistant modifiers, polyurethane (PU) is characterized by excellent abrasion resistance and good bonding properties, and it has been applied as coatings and adhesives in various fields such as architecture, textiles, and automobiles [34,35,36,37,38]. The single-component waterborne polyurethane (WPU) is one common PU material; it is easy to adjust its concentration in aqueous solutions, and it can solidify into a film with adjustable softness and hardness at room temperature [39,40]. Additionally, WPU exhibits strong interfacial bonding with various materials, such as ceramics and organic compounds, making it a highly promising and competitive abrasion-resistant modifier.

In this work, two types of WPU and one type of thermoplastic polyurethane are used as modifiers to prepare abrasion-resistant coatings on the surface of ceramic fiber threads using the dip-coating method [41]. The effects of the types and concentrations of modifiers on the microstructure and abrasion resistance performance of ceramic fiber threads are systematically investigated. Furthermore, the friction fracture mechanism and the abrasion resistance modification mechanism are explored.

## 2. Materials and Methods

### 2.1. Materials and Experiment Process

Ceramic fiber threads (SiO_2_ ≥ 96.0%) were purchased from Hubei Feilihua Quartz Glass Co., Ltd. (Jingzhou, China). The single-component hexamethylene diisocyanate waterborne polyurethane (HDI-WPU) and isophorone diisocyanate waterborne polyurethane (IPDI-WPU) modifiers were purchased from Hefei Hengtian New Material Technology Co., Ltd. (Hefei, China), and more information on the HDI-WPU and IPDI-WPU is depicted in Supplementary Data (Table S1, Figure S1). Diphenylmethane diisocyanate thermoplastic polyurethane (MDI-TPU) was purchased from Zibo Qimingxing New Material Incorporated Co., Ltd. (Zibo, China). The concentration of HDI-WPU, IPDI-WPU, and MDI-TPU was 30%.

In the typical synthesis process, the ceramic fiber threads were first heat-treated at 600 °C for 2 h. Second, a certain mass ratio of deionized water was added to the HDI-WPU or IPDI-WPU modifier to prepare a uniform solution (modifier concentrations of 3%, 9%, 15%, and 30%) by stirring with a magnetic stirrer for 30 min. Then, the ceramic fiber threads (1.5 m in each length) were dipped in the modified solution for 15 min and subsequently pulled out with a pulling rate of 0.5 cm/s. After solidifying and drying at room temperature, HDI-WPU or IPDI-WPU modified ceramic fiber threads were obtained. In addition, MDI-TPU modified ceramic fiber threads were prepared through the dip-coating preparation process using MDI-TPU as the modifier. 

### 2.2. Characterization

Scanning electron microscopy (SU1510, Hitachi, Tokyo, Japan, test voltage 5~15 kV) was used to characterize and analyze the surface morphology and microstructure of the ceramic fiber threads before and after modification. An electronic universal testing machine (CMT-4304, Meister Industrial Systems Co., Ltd. Shanghai, China) was used to measure the tensile strength of the ceramic fiber threads. Three sets of samples were tested in parallel for each group, and the average value was recorded. The linear density of the ceramic fiber threads before and after modification was calculated according to the examined length weighing method. Hydrophobic performance was tested and characterized using a contact angle measuring instrument (JC200005 M, Shanghai Zhongchen Digital Technology Equipment Co., Ltd. Shanghai, China).

**Figure 1:**
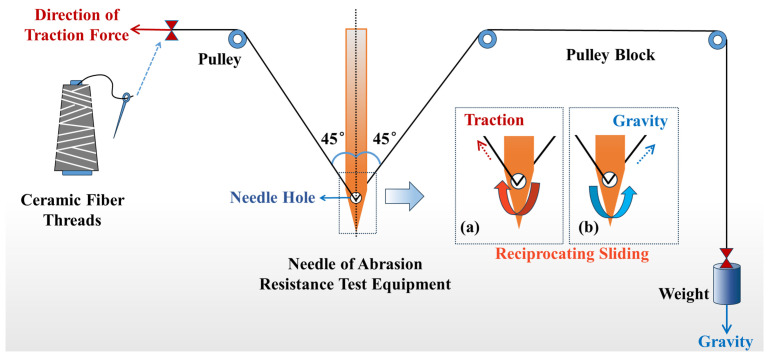
Schematic diagram of abrasion resistance performance test. Reciprocating friction motion of the ceramic fiber thread under the traction force (**a**) and the gravity of the weight (**b**).

Owing to the limited research on the abrasion resistance performance of ceramic fiber threads at specific bending angles, a set of abrasion resistance performance testing equipment was designed for testing the abrasion resistance performance of the sewing threads. This equipment can be used to evaluate the abrasion resistance performance of ceramic fiber threads at specific bending angles and to simulate the friction they undergo in practical applications. As depicted in Figure 1, the ceramic fiber thread passes through the needle hole of the test equipment, with one end connected to the traction device and the other end turned by a pulley and fixed to a weight of 25 g, forming a bending angle of 90°. During the test, the ceramic fiber thread was subjected to reciprocating friction motion under the traction force and the gravity of the weight, and the traction force was periodically applied and removed (2.5 s/time). After a certain number of friction cycles, the ceramic fiber thread fractures, and its abrasion resistance performance can be characterized by recording the number of friction cycles at the time of fracture. Three sets of samples were tested in parallel for each group, and the average value was recorded. The higher the number of friction cycles, the better the abrasion resistance performance of the ceramic fiber threads. 

## 3. Results and Discussion

### 3.1. Morphology and Structure Characterization

**Figure 2:**
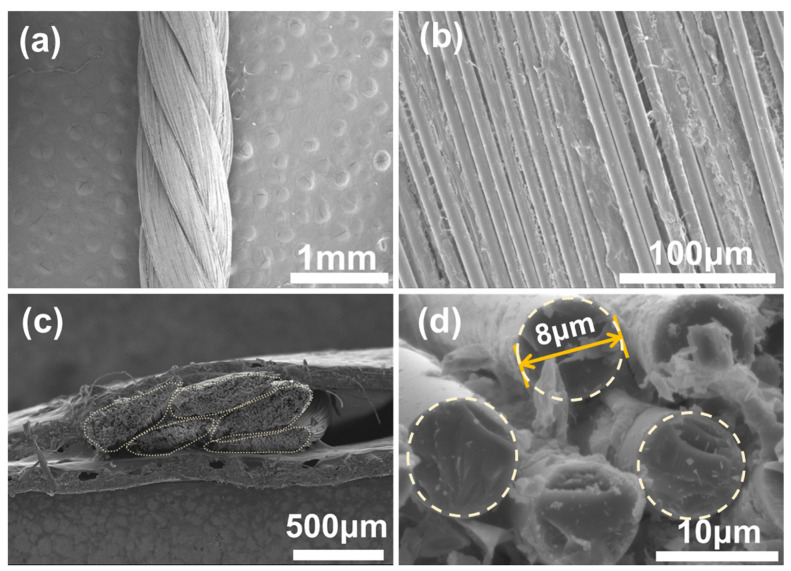
SEM images of a single original ceramic fiber thread in axial (**a**,**b**) and cross-sectional directions (**c**,**d**).

The microstructure in the axial and cross-sectional directions of the original ceramic fiber thread is shown in Figure 2a–d. It is obvious that a single ceramic fiber thread is made by twisting five strands of fiber yarn, and each yarn is composed of numerous parallel-arranged long fibers with a diameter of ~8 μm. The structure of the ceramic fiber thread is relatively loose, which corresponds to easy deformation and movement of a single yarn under external forces and good flexibility at the macro level. In addition, the surface of the fibers is covered with only a small amount of wetting agent and adjacent fibers are independent of each other, providing enough space for the fibers to undergo significant deformation when subjected to force.

**Figure 3:**
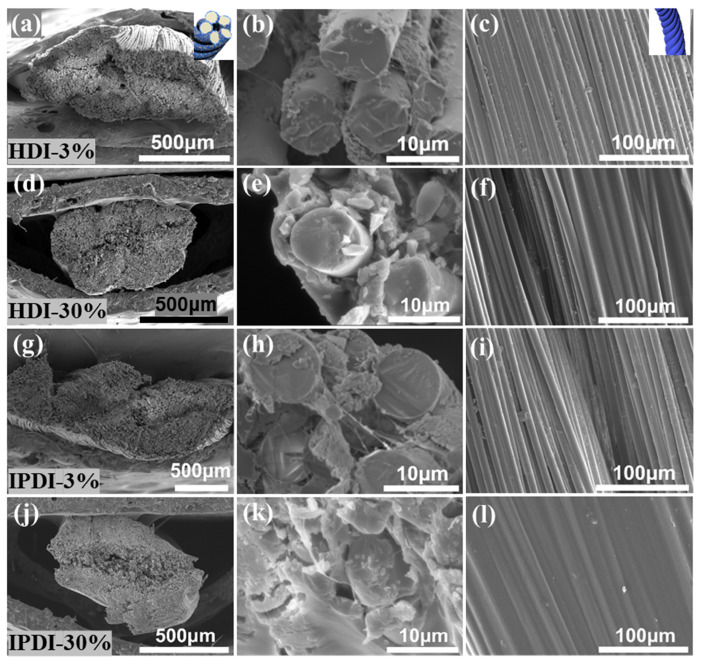
SEM images of ceramic fiber threads modified with HDI-WPU (**a**–**f**) and IPDI-WPU (**g**–**l**) at concentrations of 3% and 30%.

Figure 3a–l displays micrographs of the ceramic fiber threads modified with HDI-WPU and IPDI-WPU at concentrations of 3% and 30% in the axial and cross-sectional directions. The microstructures of the modified ceramic fiber threads at concentrations of 9% and 15%, and more details of the structure at the macro level, are depicted in Supplementary Data (Figures S2–S4). Compared with the original ceramic fiber thread, the overall structure of the HDI-WPU-modified ceramic fiber threads, composed of higher strand-integrity fibers, is relatively denser. When the modifier concentration is 3%, the HDI-WPU-modified coatings uniformly but discontinuously envelop the surface of the fibers and slightly fill some gaps between the fibers, demonstrating that the HDI-WPU features good surface wetting and interfacial bonding with the ceramic fiber threads. As the concentration of the modifier increases, the coating thickness also increases, the degree of fiber bundle densification becomes higher, and the gap-filling effect of the coating is more significant. When the modifier concentration is 30%, the coatings fill the gaps between the fibers and cover the ceramic fiber threads completely. The adjacent fibers are bonded and fixed into a whole, which greatly enhances the clustering of the ceramic fiber threads. As a result, the deformability and bending performance of the ceramic fiber threads are reduced, and the hardness of the ceramic fiber threads increases with the increase in the modifier concentration. The coating structure of the IPDI-WPU-modified ceramic fiber threads closely resembles the structure of the HDI-WPU-modified ceramic fiber threads, and the variation patterns are also similar as the concentration increases. Compared with the HDI-WPU-modified coating, the gap-filling effect of the IPDI-WPU-modified coating is more distinct, and the strand integrity of the fibers is relatively higher, corresponding to the higher hardness and decreased bending resistance. 

**Figure 4:**
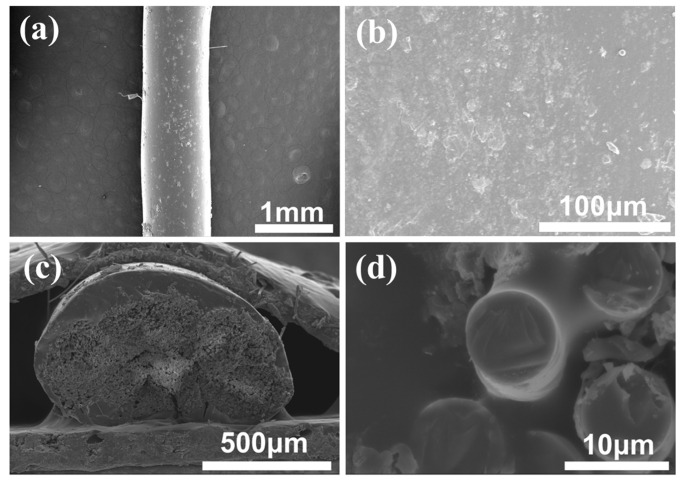
(**a**–**d**) SEM images of ceramic fiber thread modified with MDI-TPU.

MDI-TPU is one nonaqueous thermoplastic polyurethane, and the microstructure of the ceramic fiber thread modified with MDI-TPU is shown in Figure 4a–d. The coating structure of the MDI-TPU exhibits significant differences from the coating structure of the other two types of WPU. The MDI-TPU coating completely covers the ceramic fiber thread and creates a thick, nonuniform coating outside of the ceramic fiber thread, which firmly binds the fibers. Due to insufficient space to release stress through large deformation, the bending performance of the MDI-TPU-modified ceramic fiber threads is less than satisfactory.

**Figure 5:**
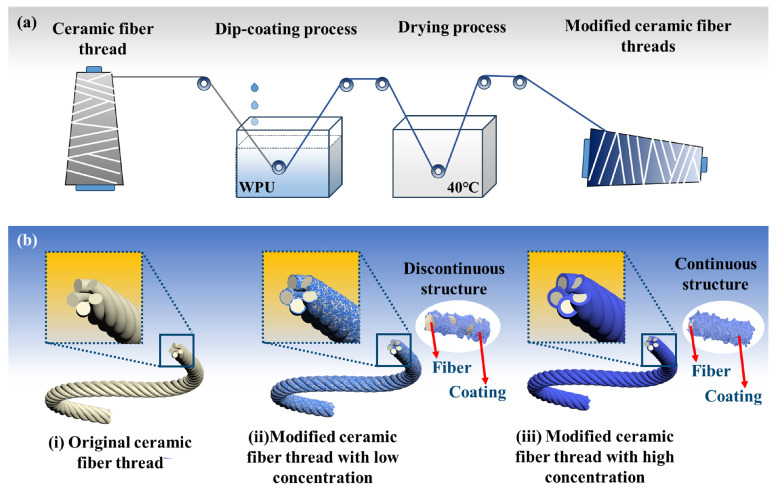
The fabrication process (**a**) and the formation mechanism of the structure of WPU-modified ceramic fiber threads (**b**).

Based on the SEM results, the fabrication process and the potential formation mechanism of the structure of WPU-modified ceramic fiber threads are shown in Figure 5a,b. Before modification, the surface of the original ceramic fiber thread is relatively bare and smooth. After modification with low-concentration WPU, the WPU adheres and solidifies on the fiber surface, and a discontinuous coating begins to form on the surface of the ceramic fiber threads. As the concentration of the modifier increases, more WPU adheres and solidifies on the fiber surface while the coating structure gradually becomes continuous, ultimately forming a continuous coating on the surface of the ceramic fiber threads.

### 3.2. Abrasion Resistance Property of Modified Ceramic Fiber Threads

**Figure 6:**
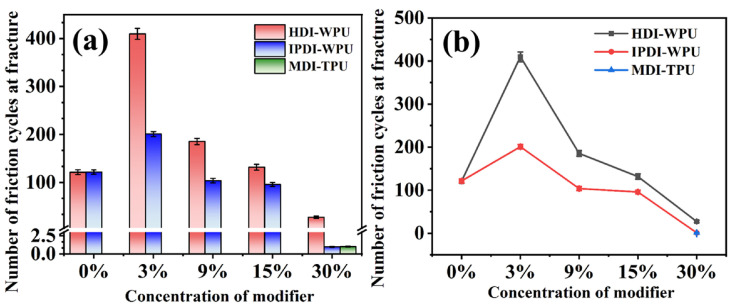
Histogram (**a**) and line chart (**b**) of the number of friction cycles at fracture of ceramic fiber threads modified with HDI-WPU, IPDI-WPU, and MDI-TPU at different concentrations.

The abrasion resistance performance test results are shown in Figure 6a,b. The number of friction cycles at fracture of the original ceramic fiber threads is only 121. After modification with HDI-WPU at a concentration of 3%, the number of friction cycles at fracture increases to 410, more than 3 times that of the original ceramic fiber threads, indicating a significant improvement in the abrasion resistance performance of the ceramic fiber threads. However, with the increase in the modifier concentration, the abrasion resistance performance shows a decreasing trend, which is attributed to the significant impact of the bending performance of the threads on the abrasion resistance performance test results. When the concentration increases, the coating fills the gaps between the fibers, and the fibers are firmly bound and fixed by the coating. The lack of sufficient space for deformation leads to a decrease in the deformability of the ceramic fiber threads. Thus, when subjected to external loads, it is difficult to adapt to bending motion through deformation. Similarly, owing to the poor bending resistance of the MDI-TPU-modified ceramic fiber threads, the abrasion resistance performance under bending angle testing is not satisfactory. After modification with IPDI-WPU, the abrasion resistance performance of ceramic fiber threads has also been greatly improved. When the modifier concentration is 3%, the number of friction cycles at fracture is 200, which is approximately twice that of the original ceramic fiber threads. Results reveal that both HDI-WPU and IPDI-WPU coatings can enhance the abrasion resistance performance of ceramic fiber threads. When the concentration is 3%, HDI-WPU exhibits an outstanding abrasion resistance modification effect.

To study the mechanism of the abrasion resistance modification, the friction fracture process and the final fracture state of the ceramic fiber threads were recorded from a macroscopic perspective. In addition, from a microscopic perspective, the microstructures of the worn area of the ceramic fiber threads before and after modification were also studied.

**Figure 7:**
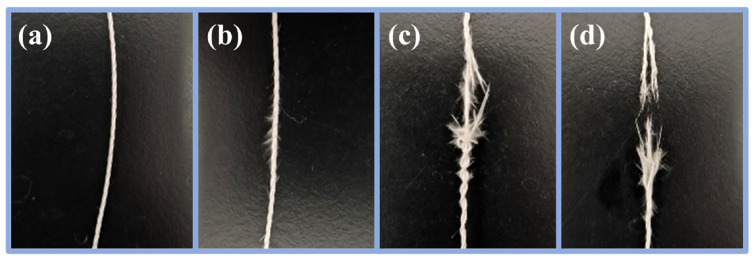
(**a**–**d**) Photographs of the ceramic fiber thread during friction fracture process.

**Figure 8:**
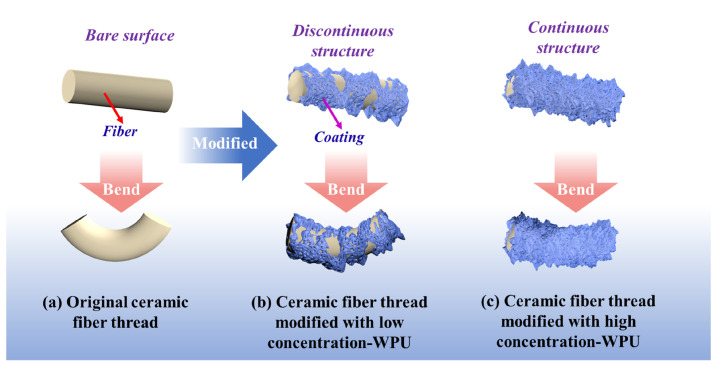
The bending and deformation mechanism of the original ceramic fiber threads (**a**), ceramic fiber threads modified with low-concentration WPU (**b**), and ceramic fiber threads modified with high-concentration WPU (**c**).

Macroscopic photographs of the ceramic fiber thread during a typical fracture process were recorded as shown in Figure 7a–d. Results suggest that the fracture process of the ceramic fiber threads mainly consists of four stages during abrasion resistance performance testing. The first stage is the bending of the ceramic fiber threads. At this stage, owing to the presence of the bending angle designed in the testing equipment, the ceramic fiber threads initially undergo bending under the combined action of tensile stress and friction. As shown in Figure 8a–c, the surface of the fibers in the original ceramic fiber threads is bare and smooth, and the deformation of the fibers is almost unhindered. Therefore, the fibers easily adapt to large deformations during bending and do not break. After low-concentration WPU modification, a unique discontinuous WPU coating is formed on the surface of the fibers, which contributes to the low resistance force during the bending deformation of the fibers. In the area without a covering coating, enough space can still be provided for the fibers to undergo large deformations, efficiently ensuring the good deformability of the fibers. However, as the concentration of the modifier increases, the coating structure gradually changes from discontinuous to continuous. The surface of the fibers is completely covered by the coating, and the gaps between fibers are also filled with the coating. These continuous coatings not only limit the deformation of the individual fibers, but also bond and fix adjacent fibers to make the ceramic fiber threads a whole. When the ceramic fiber thread is subjected to force bending, this causes more severe stress concentration, making it difficult for it to adapt to large bending angles. Therefore, good abrasion resistance performance under a bending angle requires deformability and elasticity of the ceramic fiber threads. If they cannot adapt to large-angle bends, they are prone to fracture failure at this stage.

The second stage involves the friction and fuzzing of the ceramic fiber threads. When ceramic fiber threads undergo multiple friction cycles, surface fibers will move and deform under the effect of the frictional force. Due to the lack of sufficient protection and strength, fibers ultimately aggregate and break in the local region. As the friction process continues, parts of the broken fibers will detach from the main body of the ceramic fiber thread, resulting in friction losses. The remaining fibers after fracture will continue to be subjected to friction at the fracture site, forming a weak zone and leading to the fracture of fiber bundles over a larger area. This process damages the structure of the ceramic fiber threads, leading to microscopic friction fracture and fuzzing.

The third stage is the fracture of the single strand of the ceramic fiber thread. Ceramic fiber thread is fabricated through the twisting of multiple strands of fiber yarns. After multiple cycles of friction, many fibers deform, move, and break, resulting in extensive friction fracture and fuzzing. When the cumulative friction damage reaches a certain extent, the fracture of a single strand of fiber yarn will occur.

The fourth stage involves the overall fracture of the ceramic fiber threads. During the friction process, the ceramic fiber thread was continuously subjected to the above three stages. When all of the strands of fiber yarns that made up the ceramic fiber thread broke, the ceramic fiber thread ultimately underwent overall fracture failure.

**Figure 9:**
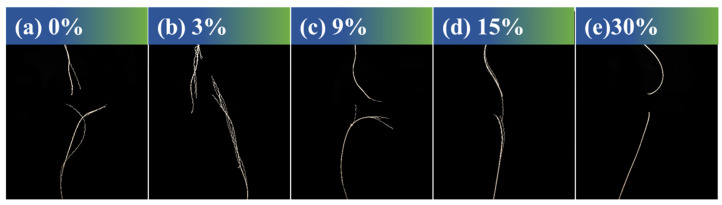
Photographs of the friction fracture area of the original ceramic fiber threads (**a**) and HDI-WPU-modified ceramic fiber threads with different concentrations (**b**–**e**).

According to the digital photos of the friction fracture areas (Figure 9 and Figure S5) of the original ceramic fiber threads and the ceramic fiber threads modified with HDI-WPU and IPDI-WPU, with increasing concentration, the ceramic fiber threads modified with low concentrations display characteristics of multiple fiber bundles with segmented ductile fracture. Moreover, the ceramic fiber threads gradually transition from strand-wise ductile fracture at low concentrations to brittle fracture along their entire length, leading to smoother fracture surfaces. This transition is attributable to the strong strand integrity of the fibers at higher concentrations. The fracture of the high-concentration ceramic fiber threads typically does not involve the third stage of strand fracture, leading directly to a one-time fracture of the entire fiber.

**Figure 10:**
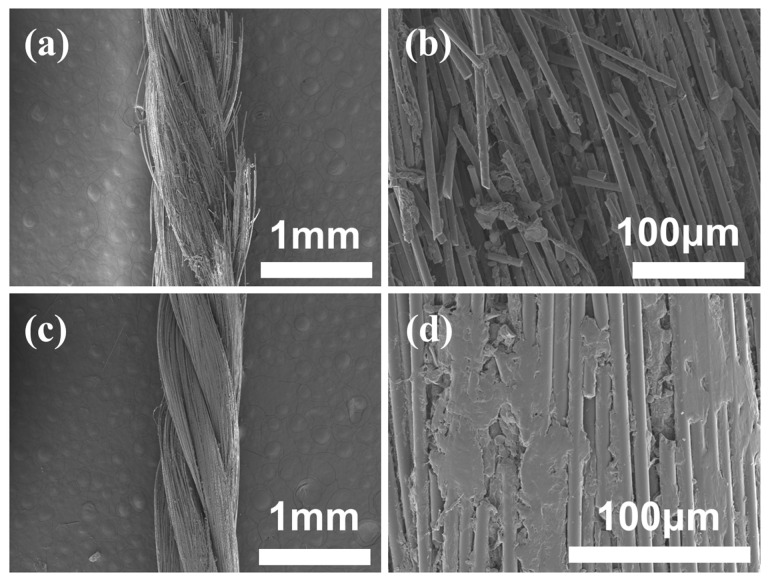
(**a**,**b**) SEM photos of the abrasion area of the original ceramic fiber thread after 100 cycles of friction. (**c**,**d**) SEM photos of the abrasion area of the HDI-WPU-modified ceramic fiber thread at a concentration of 3% after 300 cycles of friction.

Figure 10a,b presents SEM images of the abrasion area in the unmodified original ceramic fiber thread subjected to 100 cycles of reciprocating friction. The original ceramic fiber thread exhibits pronounced fracture and fuzzing on its surface, with a substantial number of fibers breaking and being pulled out. Moreover, the modifier coating exhibits a small range of slip and enrichment. Figure 10c,d shows SEM images of the abrasion area in the HDI-WPU-modified ceramic fiber thread at a concentration of 3% subjected to 300 cycles of reciprocating friction. The HDI-WPU-modified ceramic fiber thread exhibits no apparent galling, and there are hardly any observed fiber fractures. The HDI-WPU coating exhibits a broad range of slip and enrichment on the surface and makes the friction area surface smooth and dense, which contributes to effectively enhancing the abrasion resistance performance of the ceramic fiber thread.

**Figure 11:**
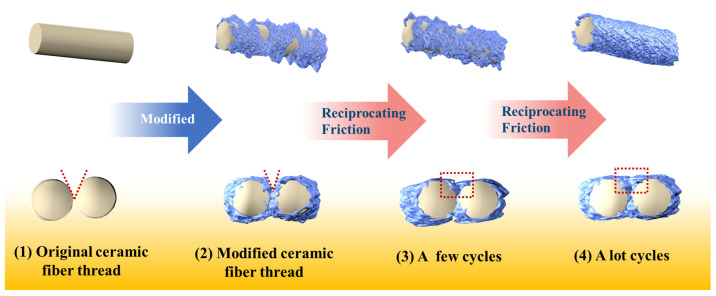
Abrasion resistance modification mechanism.

Based on the above results, we propose a potential abrasion resistance modification mechanism to explain the crucial role of the WPU coating in improving the abrasion resistance performance of ceramic fiber threads (Figure 11). The WPU coating on the surface of the ceramic fiber threads primarily serves two functions. The first function is the abrasion resistance protection. The WPU coating with good abrasion resistance performance creates a barrier between the friction surface and the fibers during the friction process, effectively prevents direct friction and abrasion on the fiber, and reduces and delays fiber friction damage. In addition, due to the strong interfacial bonding strength with the fibers, the coating can firmly adhere to the fibers and resist detachment. It provides long-term protection to the fibers, and its good elasticity and discontinuous structure contribute to adapting to the bending of the fibers, which plays a crucial role in reducing the fracture and fuzzing phenomena of ceramic fiber threads.

The second function is the lubrication effect. The WPU coating fills a few pores between the fibers and slightly reduces the macroscopic roughness of the ceramic fiber threads, which contributes to smoothing the surface of the ceramic fiber thread and reducing its surface friction coefficient. During the reciprocating friction process, the coatings gradually accumulate in the worn area of the ceramic fiber thread through sliding deformation and gradually flatten, further contributing to smoothing the surface of the ceramic fiber thread and providing an effective lubrication effect. This is equivalent to a targeted increase in the local thickness of the abrasion-resistant coating, which can further improve the abrasion resistance performance of ceramic fiber threads.

In summary, the WPU coating offers excellent protection and lubrication effects, ultimately enhancing the abrasion resistance performance of the ceramic fiber threads.

### 3.3. Tensile Property, Hydrophobicity, and Linear Density

**Figure 12:**
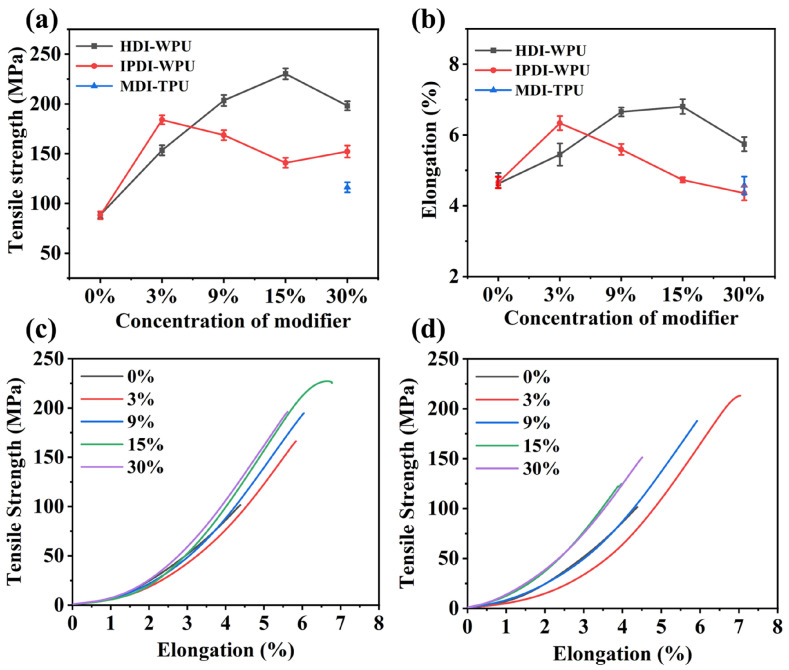
(**a**) Tensile strength and (**b**) elongation of ceramic fiber threads before and after modification. The tensile strength–elongation curves of ceramic fiber threads before and after modification with (**c**) HDI-WPU and (**d**) IPDI-WPU.

In addition to the abrasion resistance performance of the ceramic fiber threads, other key properties such as tensile strength, linear density, and hydrophobicity of the ceramic fiber threads were investigated. Figure 12a–d presents the tensile strength and elongation at fracture of the original ceramic fiber threads and the ceramic fiber threads modified with different types and concentrations of modifiers. Compared with the original ceramic fiber threads, the tensile strength of all the modified ceramic fiber threads is significantly improved. For ceramic fiber threads modified with HDI-WPU at a concentration of 3%, the tensile strength is 154.1 MPa, which is over 1.5 times the tensile strength of the original ceramic fiber thread (88.2 MPa). As the modifier concentration increases, the tensile strength and elongation first increase and then decrease. When the concentration reaches 15%, the ceramic fiber thread modified with the HDI-WPU shows the highest tensile strength, up to 229.9 MPa, and the largest elongation, of 6.9%. For ceramic fiber threads modified with IPDI-WPU, when the concentration is 3%, the tensile strength is the highest, up to 184.4 MPa. With an increase in the modifier concentration, the tensile strength shows a decreasing tendency. For ceramic fiber threads modified with MDI-TPU, the tensile strength is only 115.6 MPa. Thus, the ceramic fiber thread modified with the HDI-WPU at a concentration of 3% exhibits excellent tensile strength. 

**Figure 13:**
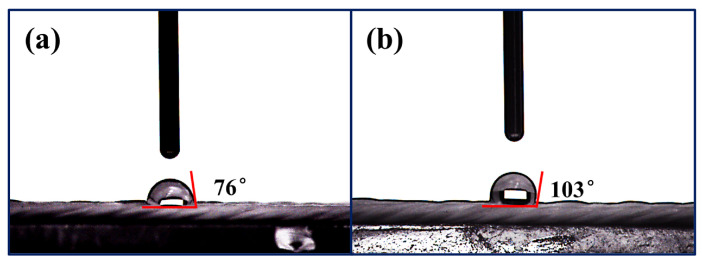
Contact angle test photos of (**a**) original ceramic fiber thread and (**b**) HDI-WPU-modified ceramic fiber thread at a concentration of 3%.

Considering the significant influence of hydrophobicity on abrasion resistance [42,43], testing the hydrophobicity of ceramic fiber threads is crucial. Therefore, the hydrophobicity of the original and the HDI-WPU-modified ceramic fiber threads was also investigated, as shown in Figure 13a,b. The contact angle of the original ceramic fiber thread is approximately 76°, whereas that of the HDI-WPU-modified ceramic fiber thread is approximately 103°, indicating that the coating also enhances the hydrophobicity of the ceramic fiber thread. The increase in hydrophobicity can be related to the roughness of the modified fiber surface.

As indicated in Table 1, HDI-WPU with a concentration of 3% significantly improves the abrasion resistance and tensile performance of the ceramic fiber thread by 3 and over 1.5 times, respectively, whereas the linear density of the ceramic fiber thread increases by only 0.3%. Results show that the HDI-WPU-modified ceramic fiber thread exhibits outstanding comprehensive performance. In addition, reduced consumption and multiple reuses constitute better choices for reusable products [44]. In this work, ceramic fiber thread, as one of the key components in flexible external thermal insulation blankets, is a reusable material. The abrasion resistance performance of ceramic fiber threads under bending conditions has a significant impact on the service life and reusable time of ceramic fiber threads. Based on the test results, the WPU modifier has excellent protective effect and abrasion resistance performance, which can significantly reduce the structural and performance damage to the fibers in the ceramic fiber threads during reciprocating friction motion, which is beneficial for extending the service life and improving reusable performance of ceramic fiber threads. Meanwhile, the tensile performance also plays an important role in the service life and reusability of ceramic fiber threads. According to the tensile performance test results, the tensile performance of the WPU-modified ceramic fiber threads is greatly improved. Thus, the WPU modifier is beneficial for extending service life and increasing reusable time, and it might have effects on the life-cycle analysis of ceramic fiber threads in terms of the end-of-life aspect.

## 4. Conclusions

In summary, a novel WPU abrasion-resistant coating is fabricated on the surface of ceramic fiber threads using the dip-coating method, and the effects of the modifier type and concentration on the microstructure and abrasion-resistant property under a certain bending angle of the ceramic fiber threads are investigated. Among the three modifiers, both the HDI-WPU and IPDI-WPU modifiers can efficiently enhance the abrasion resistance performance of ceramic fiber threads and adapt to the large bending angle. The discontinuous structure of the coatings is crucial for the deformability and bending capacity of the ceramic fiber threads. When the modifier concentration is 3%, the abrasion resistance performance of the HDI-WPU-modified ceramic fiber thread (the number of friction cycles at fracture of 410) is enhanced by over three times, and the tensile performance (tensile strength of 154.1 MPa) is improved by over one and a half times. Simultaneously, the linear density of the HDI-WPU-modified ceramic fiber thread is only 0.3% higher than that of the original ceramic fiber thread, indicating that the HDI-WPU-modified ceramic fiber thread possesses an outstanding comprehensive performance. Moreover, the hydrophobic property of the modified thread is also enhanced. During abrasion resistance performance testing, the WPU coating plays an important role in creating a protective barrier and lubrication to enhance the abrasion resistance performance of the product. The results suggest that introducing HDI-WPU onto the surface of ceramic fiber threads to form a coating is a highly promising strategy for efficiently enhancing the abrasion resistance performance of ceramic fiber threads subjected to a specific bending angle.

## Figures and Tables

**Table 1:** Comprehensive performance of ceramic fiber threads before and after modification.

Type of Modifier	Concentration (%)	Linear Density (Tex)	Friction Cycles at Fracture	Tensile Strength (MPa)	Elongation (%)
HDI-WPU	3	461	410	154.1	5.5
	9	472	186	203.9	6.6
	15	479	131	229.9	6.9
	30	514	28	197.8	5.7
IPDI-WPU	3	467	200	184.4	6.3
	9	478	103	168.7	5.6
	15	500	96	140.2	4.8
	30	544	1	152.3	4.4
MDI-TPU	/	772	1	115.6	4.5
Without modifier	0	460	121	88.2	3.9

## Data Availability

Data are contained within the article and supplementary materials.

## Supplementary Materials

The following supporting information can be downloaded at: https://www.mdpi.com/article/10.3390/ma17030599/s1, Table S1: More details of HDI-WPU and IPDI-WPU; Figure S1: Particle size distributions of HDI-WPU dispersion (a) and IPDI-WPU dispersion (b); Figure S2: SEM images of ceramic fiber threads modified with HDI-WPU (a)–(f) and IPDI-WPU (g)–(l) at concentrations of 9% and 15%; Figure S3: SEM images of ceramic fiber threads modified with HDI-WPU at concentrations of (a) 3%, (b) 9%, (c) 15%, and (d) 30%; Figure S4: SEM images of ceramic fiber threads modified with IPDI-WPU at concentrations of (a) 3%, (b) 9%, (c) 15%, and (d) 30%; Figure S5: Photographs of the friction fracture area of the IPDI-WPU-modified ceramic fiber threads with concentrations of (a) 3%, (b) 9%, (c) 15%, and (d) 30%.
