# Supplementary material for: Preparation of Ceramic Fiber Threads with Enhanced Abrasion Resistance Performance
Source: Materials (Basel). 2024 Jan 26;17(3):599. doi: 10.3390/ma17030599 (PMC10856284; doi:10.3390/ma17030599)
Supplement: Supplementary file 1 [file materials-17-00599-s001.zip › materials-2804984-supplementary.pdf]

materials

Supporting Information

Preparation of Ceramic Fiber Threads with Enhanced Abrasion Resistance Performance

Xueying Zhang, Feng Hou *, Haiyan Du, Liwen Yan, Anran Guo, Xiaohui Ma and Jiachen Liu *

Key Laboratory of Advanced Ceramics and Machining Technology of Ministry of Education, School of Materials Science and Engineering, Tianjin University, Tianjin 300072, China; zxy_92@163.com (X.Z.); hydu@tju.edu.cn (H.D.); lwyan@tju.edu.cn (L.Y.); arguo@tju.edu.cn (A.G.); xiaohuima@tju.edu.cn (X.M.)

* Correspondence: houf@tju.edu.cn (F.H.); jcliutju@tju.edu.cn (J.L.)

Table S1. More details of HDI-WPU and IPDI-WPU.

Type of modifier	Concentration (%)	Particle size d_{50} (μm)	Tensile strength (MPa)	Elongation (%)
HDI-WPU	30	0.110	＞20	＞900
IPDI-WPU	30	0.108	＞15	＞700

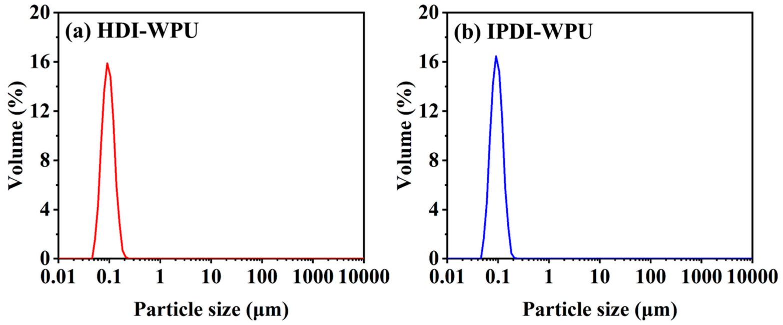

Figure S1. Particle size distributions of HDI-WPU dispersion(a) and IPDI-WPU dispersion(b).

The particle size and distribution of the HDI-WPU and IPDI-WPU particles were measured and analyzed by a laser particle analyzer (Malvern 2000, UK) at ambient temperature.

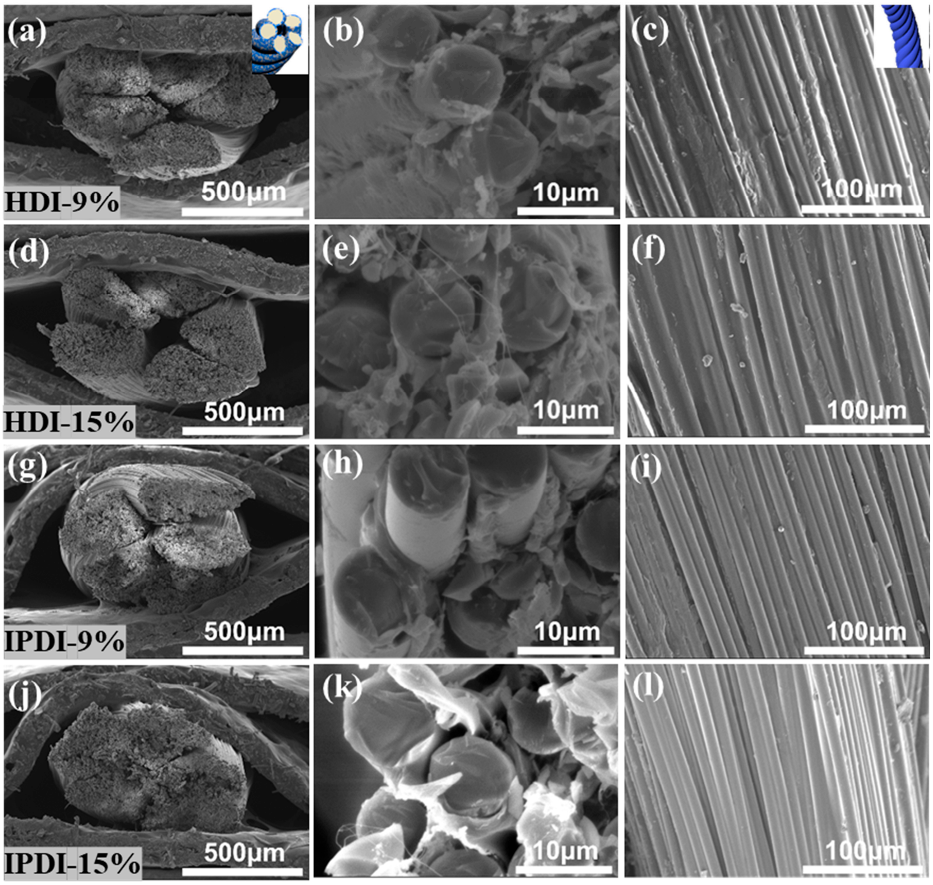

Figure S2. SEM images of ceramic fiber threads modified with HDI-WPU (a)-(f) and IPDI-WPU (g)-(l) at concentrations of 9% and 15%.

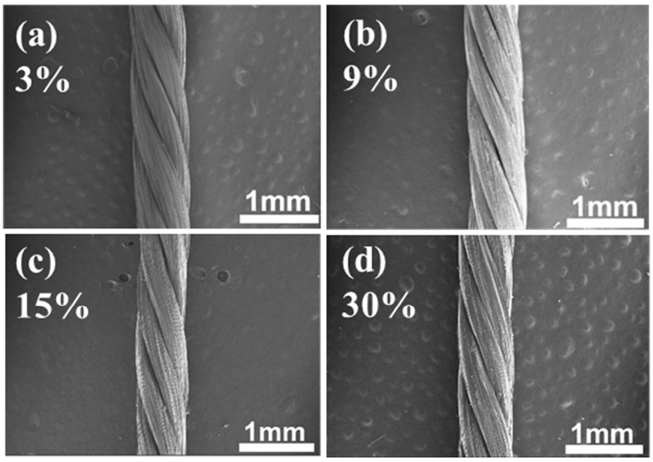

Figure S3. SEM images of ceramic fiber threads modified with HDI-WPU at concentrations of (a) 3%, (b) 9%, (c) 15% and (d) 30%.

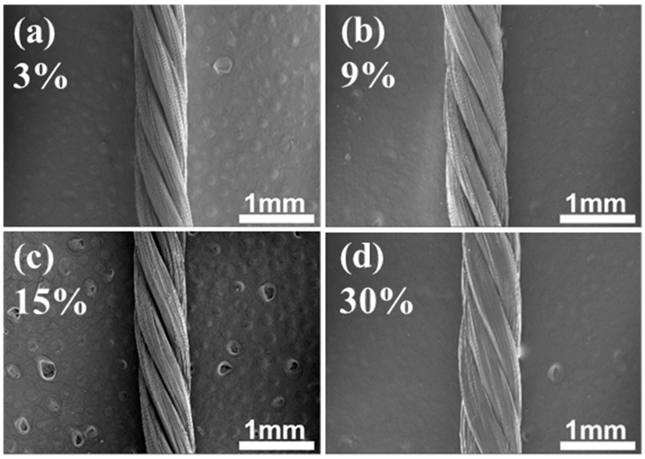

Figure S4. SEM images of ceramic fiber threads modified with IPDI-WPU at concentrations of (a) 3%, (b) 9%, (c) 15% and (d) 30%.

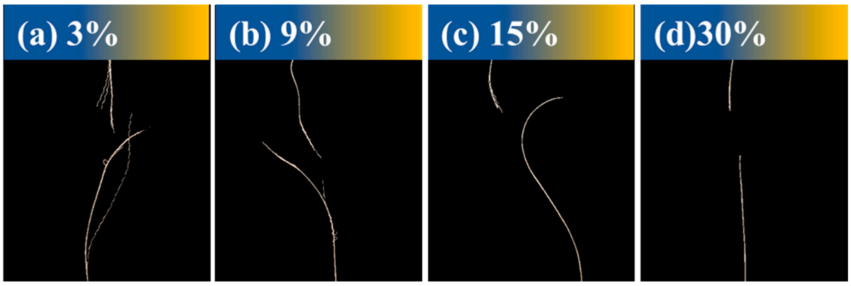

Figure S5. Photographs of the friction fracture area of the IPDI-WPU-modified ceramic fiber threads with concentrations of (a) 3%, (b) 9%, (c) 15% and (d) 30%.
